# Elimination of intravascular thrombi prevents early mortality and reduces gliosis in hyper-inflammatory experimental cerebral malaria

**DOI:** 10.1186/s12974-018-1207-4

**Published:** 2018-06-04

**Authors:** Kyle D. Wilson, Lorenzo F. Ochoa, Olivia D. Solomon, Rahul Pal, Sandra M. Cardona, Victor H. Carpio, Philip H. Keiser, Astrid E. Cardona, Gracie Vargas, Robin Stephens

**Affiliations:** 1 Department of Microbiology and Immunology, University of Texas Medical Branch, 301 University Boulevard, Galveston, TX 77555 USA; 2 Center for Biomedical Engineering, University of Texas Medical Branch, 301 University Boulevard, Galveston, TX 77555 USA; 3 Department of Biology, One UTSA Circle, University of Texas at San Antonio, San Antonio, TX 78249 USA; 4 Department of Neuroscience and Cell Biology, University of Texas Medical Branch, 301 University Boulevard, Galveston, TX 77555 USA; 5 Department of Internal Medicine, Division of Infectious Diseases, University of Texas Medical Branch, 301 University Boulevard, Galveston, TX 77555-0435 USA; 6 Institute for Human Infections and Immunity, University of Texas Medical Branch, 301 University Boulevard, Galveston, TX 77555 USA

**Keywords:** Malaria, Brain, Inflammation, Neuropathology, Vascular congestion, Monocyte, Astrocyte, Microglia, Immunofluorescence, Confocal microscopy

## Abstract

**Background:**

Cerebral malaria (CM) is the most lethal outcome of *Plasmodium* infection. There are clear correlations between expression of inflammatory cytokines, severe coagulopathies, and mortality in human CM. However, the mechanisms intertwining the coagulation and inflammation pathways, and their roles in CM, are only beginning to be understood. In mice with T cells deficient in the regulatory cytokine IL-10 (IL-10 KO), infection with *Plasmodium chabaudi* leads to a hyper-inflammatory response and lethal outcome that can be prevented by anti-TNF treatment. However, inflammatory T cells are adherent within the vasculature and not present in the brain parenchyma, suggesting a novel form of cerebral inflammation. We have previously documented behavioral dysfunction and microglial activation in infected IL-10 KO animals suggestive of neurological involvement driven by inflammation. In order to understand the relationship of intravascular inflammation to parenchymal dysfunction, we studied the congestion of vessels with leukocytes and fibrin(ogen) and the relationship of glial cell activation to congested vessels in the brains of *P. chabaudi*-infected IL-10 KO mice.

**Methods:**

Using immunofluorescence microscopy, we describe severe thrombotic congestion in these animals. We stained for immune cell surface markers (CD45, CD11b, CD4), fibrin(ogen), microglia (Iba-1), and astrocytes (GFAP) in the brain at the peak of behavioral symptoms. Finally, we investigated the roles of inflammatory cytokine tumor necrosis factor (TNF) and coagulation on the pathology observed using neutralizing antibodies and low-molecular weight heparin to inhibit both inflammation and coagulation, respectively.

**Results:**

Many blood vessels in the brain were congested with thrombi containing adherent leukocytes, including CD4 T cells and monocytes. Despite containment of the pathogen and leukocytes within the vasculature, activated microglia and astrocytes were prevalent in the parenchyma, particularly clustered near vessels with thrombi. Neutralization of TNF, or the coagulation cascade, significantly reduced both thrombus formation and gliosis in *P. chabaudi*-infected IL-10 KO mice.

**Conclusions:**

These findings support the contribution of cytokines, coagulation, and leukocytes within the brain vasculature to neuropathology in malaria infection. Strikingly, localization of inflammatory leukocytes within intravascular clots suggests a mechanism for interaction between the two cascades by which cytokines drive local inflammation without considerable cellular infiltration into the brain parenchyma.

**Electronic supplementary material:**

The online version of this article (10.1186/s12974-018-1207-4) contains supplementary material, which is available to authorized users.

## Background

With 212 million new cases and 429,000 estimated deaths in 2015, malaria remains one of the most economically impactful infectious diseases worldwide [1]. A small percentage of *Plasmodium falciparum* infections results in severe malarial disease. However, a significant proportion of severe malaria infections includes cerebral malaria (CM), which is a leading cause of death in sub-Saharan African children and represents a major burden worldwide [2]. CM accounts for an estimated 500,000 cases per year and correlates with high parasitemic burden, severe inflammation, and cerebral edema [2]. Furthermore, about 20% of patients with CM die despite timely treatment [3], and neurological sequelae in survivors is common [4]. Several host genetic factors have been implicated in pathology. For example, mutations in the promoters of the inflammatory cytokine tumor necrosis factor (TNF), which drives the anti-malaria response of phagocytes, and the regulatory cytokine IL-10, which protects the host from excessive immunopathology, have been correlated with severe disease in both mice and humans [5–10]. However, inflammatory cytokines also allow parasite sequestration and leukocyte adhesion by upregulating adhesion molecules on the vascular endothelium [11–13].

The role of inflammatory cytokines increased by the absence of IL-10 has been studied extensively in the *Plasmodium chabaudi* mouse model of severe malaria [14]. *P. chabaudi* is a rodent parasite that leads to mild malaria in C57BL/6 (WT) mice. However, in IL-10-deficient (IL-10 KO) mice, *P. chabaudi* infection leads to hyper-inflammation and death. The syndrome includes increased levels of the pro-inflammatory cytokines TNF and IFN-γ [14] and lethal disease characterized by cerebral pathology including cerebral edema and hemorrhage [15]. In addition, we have recently demonstrated pathological behavioral phenotypes indicative of neurological and cognitive dysfunction in this model [16]. Strikingly, there is no significant parasite sequestration in the brains of these mice. While a few parasites have been detected in the brain vasculature via electron microscopy [17], a more recent examination of the brain using highly sensitive luminescence technology to detect luciferin-expressing *P. chabaudi* parasites did not show significant enrichment [18]. The *P. chabaudi* life cycle is synchronous. Mature schizonts disappear from the circulation almost completely and are found sequestered primarily in the liver and lungs of mice in a partially ICAM1-dependent manner [19]. Interestingly, pathological damage within each organ in *P. chabaudi* does not correspond to the degree of organ-specific sequestration of the parasite [18]. Sequestration is a hallmark of autopsy in fatal *P. falciparum*-induced CM cases [20, 21], and specific parasite variants are associated with severe malaria [22–25]; however, it is challenging to definitively prove that parasite sequestration in the brain is causal to CM.

Activated immune cells and pro-inflammatory cytokines are also strongly implicated in the mortality in human disease [26, 27]. A low ratio of IL-10 to TNF in patients predicts more severe malaria, as do mutations in the IL-10 and TNF genes [28, 29]. Mouse models show that this is because IL-10 is required to protect animals from lethal pathology, as it regulates the pro-inflammatory cytokines IL-12 and TNF [30], which drive as yet poorly defined neuroimmunopathology. IL-10 KO mice lacking IFN-γ receptor signaling are also rescued from mortality, even though they exhibit higher levels of parasitemia [31]. IL-10 is primarily made by CD4^+^ IFN-γ^+^ effector T cells (Teff) in *P. chabaudi* infection, not Tregs, and is downstream of IL-27 [32, 33], and we have shown that CD4 Teff are found solely within the cerebral vasculature, not in the brain parenchyma [16].

While there are studies of host genetics and those correlating systemic inflammatory cytokines with poor outcomes in severe malaria [26, 27], no significant inflammatory infiltrate within the brain parenchyma has been documented in human or mouse studies of the disease [20, 21, 34–40]. As a result, the contribution of activated peripheral leukocytes to brain pathology has been poorly appreciated. Interestingly, despite the lack of infiltrating inflammatory cells in the brain parenchyma, we have documented increased microglial activation in this model [16]. This is intriguing because glia are found behind the multi-layered blood-brain barrier (BBB), while activated peripheral immune cells are within the vasculature [16]. This prompted the question of how the inflammatory cells within the vasculature could amplify cytokine production in the absence of a lymphoid structure, such as that developing in neuroimmunopathologies with parenchymal infiltrates.

Congestion of the brain and retinal vasculature has been documented in human cerebral malaria and is associated with poor prognoses in human cases of CM [41, 42]. Several factors are likely to contribute to congestion in human patients: parasite sequestration, leukocyte adhesion, and coagulation defects. Parasite-infected erythrocytes can both bind to the vascular endothelium, leading to activation and vascular dysfunction, and activate the coagulation cascade [43, 44]. Coagulation defects are also seen in both murine experimental cerebral malaria and in human cerebral malaria [45–47] and can be promoted by the parasite itself [45]. Vascular thrombi were observed in CM2 patients in Malawi, who are documented to have both sequestration and cerebral hemorrhages [20]. This supports the finding that disseminated intravascular coagulation (DIC) was observed in 19% of CM patients and correlated with poor outcomes [48]. However, the role of coagulation in neuropathology is obscured by contradictory outcomes in studies of the effect of the anticoagulant, heparin [49, 50]. In clinical trials, heparin significantly reduced death in a clinical trial in children with CM in Indonesia (from 13/17 to 2/16, [50]) and reduced patient’s coma and hospitalization time [49]. However, it is not currently recommended for treatment due to the potential of systemic hemorrhagic side effects of this older drug, suggested by work in non-human primates [51] and case studies of malarious soldiers in Asia with pulmonary involvement [52], though not seen in clinical trials. The presence of monocytes and T cells in the brain vasculature [20], but not in the brain parenchyma [34], is also documented. This has often been interpreted as a “lack of inflammation,” despite strong evidence, both genetic and serological, that cytokines play a critical role in killing parasite and inducing pathology [53].

In an attempt to understand the role of adherent intravascular leukocytes and coagulation in promoting neuronal malfunction, we investigated the contents of congested vessels and their effects on the brain parenchyma, as measured by gliosis. Furthermore, we tested the role of coagulation in pathology by studying the effect of anticoagulants on mortality and histological features of inflammation-driven neuropathology in *P. chabaudi* infection of IL-10 KO mice. We found that thrombi were prevalent throughout the brain and coincide with localization of adherent leukocytes. In addition, areas of coagulation and leukocytes co-localized with parenchymal gliosis. We also found a striking reduction of mortality and a significantly recovered parenchymal histology on elimination of coagulation suggesting a pathological role for thrombi in this model. These observations suggest an important role of coagulation in vascular congestion in CM and also implicate a novel mechanism of inflammation-induced neuropathology possibly initiated by leukocytes contained within the vasculature. These findings may be relevant because the inflammation-driven neuropathology in this model shares many features with human cerebral malaria, including intravascular leukocytes and thrombi, systemic hyper-inflammation, edema, and death.

## Methods

### Mice

C57BL/6J (WT) and B6.129P2-Il10^tm1Cgn^/J (IL-10 KO) mice (Jackson Laboratory, Bar Harbor, ME) were bred in The University of Texas Medical Branch Animal Resource Center. Experimental mice were female and between 6 and 12 weeks of age at the time of infection. All animals were kept in a specific pathogen-free housing with ad libitum access to food and water. Animals were cared for according to the Guide for the Care and Use of Laboratory Animals under Institutional Animal Care and Use Committee-approved protocols. UTMB Animal Resource Center facilities operate in compliance with the USDA Animal Welfare Act, the Guide for the Care and Use of Laboratory Animals, under OLAW accreditation, and IACUC-approved protocols.

### Parasite and infection

Frozen stocks of *Plasmodium chabaudi chabaudi* (*AS*)-infected RBCs (iRBCs) (Jean Langhorne, Francis Crick Institute, London, UK) kept at − 80 °C were thawed and injected intraperitoneally (i.p.) into WT mice. Parasitized blood from these animals was diluted in Krebs-Ringer bicarbonate buffer (Sigma-Aldrich, St. Louis, MO) and normal saline to deliver 10^5^ iRBCs i.p. in 200 μl into experimental WT or IL-10 KO mice. Thin blood smears were collected at regular intervals to monitor for peripheral parasitemia by staining with Diff-Quik (Siemens Healthcare Diagnostics, Newark, DE) or Giemsa stain (Ricca Chemical Company, Arlington, TX) and counted on a light microscope.

### Animal body temperature and weight

Internal body temperatures were assessed daily during infection using rounded stainless steel rectal probes and a BIO-TK8851 digital rodent model thermometer (Bioseb, Pinellas Park, FL). Probes were sanitized with CaviCide (Metrex Research Corp., Romulus, MI) between each use. Animal weights were measured using an OHAUS Scout Pro SP601 portable balance (OHAUS, Parsippany, NJ).

### Animal behavior evaluation

Beginning on day 5 post-infection, daily assessments were performed on all animals using an abbreviated version of the modified SmithKline Beecham, Harwell, Imperial College, Royal London Hospital Phenotype Assessment (SHIRPA) protocol [54]. This brief behavioral assessment was developed based on the full assessment in a previous study [16]. Higher scores were awarded for measures showing higher functional ability. The procedures were carried out in an open testing environment away from the home cage and took approximately 5 min per animal.

The abbreviated SHIRPA used involves a selection of nine semi-quantitative tests for general health and sensory function, baseline behaviors, and neurological reflexes. We observed undisturbed behavior with the mouse placed in an inverted beaker on top of a metal grid suspended above the home cage for 3 min, during which body position and spontaneous activity were assessed. Body position scores ranged from 0 (completely flat) to 5 (repeated vertical leaping). Spontaneous activity scores ranged from 0 (none) to 4 (rapid/dart movement). At the end of the observation period, palpebral closure, which was scored from 0 (eyes closed) to 2 (eyes wide open), and qualitative grip strength, scored from 0 (none) to 4 (unusually strong), are tested by applying a gentle horizontal force on the animal’s tail as it grips the metal grid. The animal is then placed in an open arena in which several behaviors are measured. Gait is observed as the animal traverses the arena and is scored from 0 (incapacity) to 3 (normal). During movement, tail elevation is scored, ranging from 0 (dragging) to 2 (elevated). Touch escape measures the reaction to a finger stroke and is scored from 0 (no response) to 3 (escape response to approach). Palpation of the animal’s sternum determines heart rate: 0 (slow) to 2 (fast), and finally, righting reflex is scored by releasing the animal from an upside-down position near the surface and observing the responding effort to upright itself, scored from 0 (fails to right) to 3 (lands on feet). The expected score of a healthy, uninfected IL-10 KO or WT mouse is 22. A score of 15 was identified as the humane endpoint based on the finding that any female animal that drops below that score by day 9 will succumb to infection (see Additional file 1: Figure S1).

### Histochemistry

Immunofluorescence of cryosections was examined after 48 h of post-fixation of mouse brains in 4% PFA and 72 h of cryoprotection in 30% sucrose. Fixed frozen sagittal sections (30 μm) were made using Tissue Plus® Optimal Cutting Temperature Compound (Fisher Healthcare, Houston, TX) and mounted on glass slides with Fluoromount mounting medium (Novus Biologicals, Littleton, CO). Sections were incubated overnight at 4 °C with primary antibodies rabbit anti-fibrinogen (catalog no. A0080, Agilent Technologies, Carpinteria, CA), rat (clone 2.2B10, catalog no 13-0300, Thermo Fisher Scientific, Waltham, MA), or rabbit (catalog no. Z0334, Agilent Technologies, Carpinteria, CA) anti-GFAP, mouse anti-CD11b biotin (clone M1/70, catalog no. 13-0112-85, eBioscience, San Diego, CA), and rat anti-CD45 biotin (clone 104, catalog no. 13-0454-85, eBioscience, Sand Diego, CA). Secondary antibodies used were goat anti-rat AlexaFluor-488 (catalog no. A11006, Thermo Fisher Scientific, Waltham, MA) and goat anti-rabbit AlexaFluor-568 (catalog no. A11011, Thermo Fisher Scientific, Waltham, MA). Streptavidin-FITC (catalog no. 11-4317-87, eBioscience, San Diego, CA) was used as a tertiary step for biotinylated antibodies. CellTrace Violet (catalog no. C34557, Thermo Fisher Scientific, Waltham, MA)-labeled CD4 T cells were adoptively transferred into IL-10 KO mice for later co-localization with brain vasculature after i.v. perfusion with DyLight488-labeled tomato lectin (catalog no. DL-1174, Vector Laboratories, Burlingame, CA). Images of immunohistochemistry (IHC) sections were taken with an Olympus IX 71 inverted brightfield microscope using a × 20 air objective, while the immunofluorescence images were taken with a confocal microscope (Olympus FV 1000) with the DAPI channel for nuclei, Alexa 488 channel for Iba1 tagged with Alexa 488, and Alexa 647 channel for CD 31 tagged with Alexa 647. IHC images of Iba1-stained sections were contrast-enhanced and segmented by threshold for microglia using ImageJ (NIH, Version 1.48u). These were used to create binary images. Individual microglia were identified using a semi-automatic algorithm employing the particle analysis function on image and average area per microglia; the microglia density and total immunoreactive area were calculated from the binary images. Area fraction of small processes is a ratio of immunoreactive area without microglia to total immunoreactive area which indicates the degree of ramification. Transformation index, and indicator of activation, was calculated as T-Index = (Perimeter^2^)/(4π × Area) per microglia. To quantitatively describe the degree of ramification, we calculated the area fraction of small thin processes to total immunoreactive area. Ramification could be seen in IHC images as glia with long and thin processes that appeared segmented due to branching in and out of the tissue section plane. The astrocyte-thrombus association index was defined in which the ratio of *X*_i_ (the number of astrocytes contacting a thrombus divided by the total number of thrombi) was calculated, and values were normalized based on the following equation, (*X*_i_ − *X*_min_)/(*X*_max_ − *X*_min_), where *X*_min_ = 1.3 (lower limit of astrocyte-thrombi interaction seen in uninfected IL-10 KO brains) and *X*_max_ = 3.25 (~ 75% astrocyte/thrombi association) approximated the lower and upper limit of astrocytes interacting with thrombi based on our data.

### Cell and in vivo labeling

Some infected IL-10 KO and WT animals were injected with 2 × 10^6^ CTV^+^ CD4 T cells 3.5 h before sacrifice (i.p.) and 40 μg of DyLight488 labeled *Lycopersicon esculentum* (tomato) Lectin (catalog no. DL-1174, Vector Laboratories, Burlingame, CA) 20 min before sacrifice (i.v.). CellTrace Violet (catalog no. C34557, Thermo Fisher Scientific, Waltham, MA) labeling was performed as previously described [55].

### Anti-TNF antibody treatment

Mice receiving anti-TNF antibody (clone XT3.11, Bio X Cell, West Lebanon, NH) were treated with 0.2 μg/day for 5 days starting on day 5 post-infection (days 5–9). Untreated mice received isotype rat IgG1 as a control.

### CLARITY and optical clearing

Fixed brain sections (IL-10 KO and WT) were subjected to the passive CLARITY optical clearing method [56] for large-scale labeling and imaging. In brief, mice were anesthetized and perfused transcardially with a mixture of 4% (wt/vol) PFA, 4% (wt/vol) acrylamide, 0.05% (wt/vol) bis-acrylamide, and 0.25% (wt/vol) VA044 (hydrogel solution) in PBS. Brains were extracted and incubated in hydrogel solution at 4 °C for 3 days. Solution temperature was then increased for 3 h to 37 °C to initiate polymerization. Hydrogel-embedded brains were sectioned into 2-mm-thick sagittal sections and placed in clearing solution (sodium borate buffer, 200 mM, pH 8.5) containing 4% (wt/vol) SDS) for 3 weeks at 40 °C under gentle agitation. Samples were immunostained for GFAP to assess astrogliosis. After immunostaining, samples were optically cleared using increasing serial concentrations (10–100%) of 2,2′-thiodiethanol (TDE) in Milli-Q water (EMD Millipore, Darmstadt, Germany) to achieve optimal refractive index matching with tissue.

### Microscopy

Fixed cryosections (30 μm thickness, fluorescent or confocal microscopy) were imaged with a Nikon Eclipse 80i epifluorescence microscope and a Fluoview 1000MPE system configured with an upright BX61 microscope (Olympus, Center Valley, PA). Fixed, CLARITY-processed sections (2 mm thickness, two-photon confocal microscopy) were imaged using a Prairie Ultima IV (Prairie Technologies/Bruker, Middleton, WI) upright multiphoton microscope. For two-photon fluorescence microscopy, a × 10 0.3 N.A. objective (UPLFL10X, Olympus) and a × 25 1.05 N.A. super-objective (XLSLPLN25XGMP, Olympus) were used for image collection. Illumination for excitation of fluorescence was provided by a femtosecond laser (Mai Tai, SpectraPhysics, Santa Clara, CA) tuned to 800 nm. Fluorescence was collected using a two-photon standard M filter set including filters with bandwidth 604 ± 45 nm, a filter with bandwidth 525 ± 70 nm, and dichroic mirror cutoff at 575 nm. Samples were mounted on a 30-mm cage plate (CP06, ThorLabs, Newton, NJ) between two #1.5 cover glass. To visualize large regions of optically cleared brain tissue using two-photon microscopy, image stack mosaic and stitching were applied. Image stack stitching was done with a 10% overlap on a field of view of 2327.3 × 237.3 μm providing 232.73 μm of co-registration in *X* and *Y* coordinates. Images were analyzed using ImageJ (FIJI), Olympus Fluoview FV1000-ASW 2.0 Viewer (confocal), Imaris Image Analysis Software (confocal and two-photon microscopy; Bitplane USA, Concord, MA), and NIS Elements (confocal; Nikon Instruments, Melville, NY). Positive fibrinogen and elevated GFAP staining in each field was quantified by applying a signal intensity threshold and the percent area covered was calculated via the outlined areas of positive staining that met the signal intensity threshold per field of view. The percentage of total area included was calculated using ImageJ software (FIJI, NIH).

### Ammonia assay

Tissue and serum ammonia was quantified using a commercial colorimetric ammonia assay kit (ab83360, Abcam, Cambridge, MA). Briefly, brain and liver samples were collected from infected IL-10 KO and WT mice at the peak of behavioral symptoms, washed in cold PBS, resuspended in 100 μl assay buffer, and homogenized using a Dounce homogenizer to produce single-cell suspensions. After 2–5 min of centrifugation at 4 °C, cells were counted via hemocytometer and seeded into a 96-well plate to provide 1–5 × 10^4^ cells/well. Serum samples were counted and seeded directly into plates without processing (5–10 μl/well). The colorimetric assay was conducted using OxiRed probe. Color change was recorded at OD 570 nm using a spectrophotometer microplate reader and compared to an ammonium chloride standard curve (detects 0–10 nmol/well) after 60 min of incubation at 37 °C.

### Statistics

Where indicated, groups were compared by *t* test (2 groups) or one-way ANOVA (3 or more groups), followed by post hoc Bonferroni method or Tukey’s test to identify significance between individual groups. Each point represents the average value per animal after analysis of 10 fields, unless otherwise specified. Statistical analysis was performed in Prism (GraphPad, La Jolla, CA), *p ≤ 0.05, **p ≤ 0.01, and ***p ≤ 0.001. Error bars represent ± SEM.

## Results

### Congestion of brain blood vessels with thrombi containing CD45^+^, CD11b^+^, and CD4^+^ leukocytes in *P. chabaudi*-infected IL-10 KO mice

**Fig. 1:**
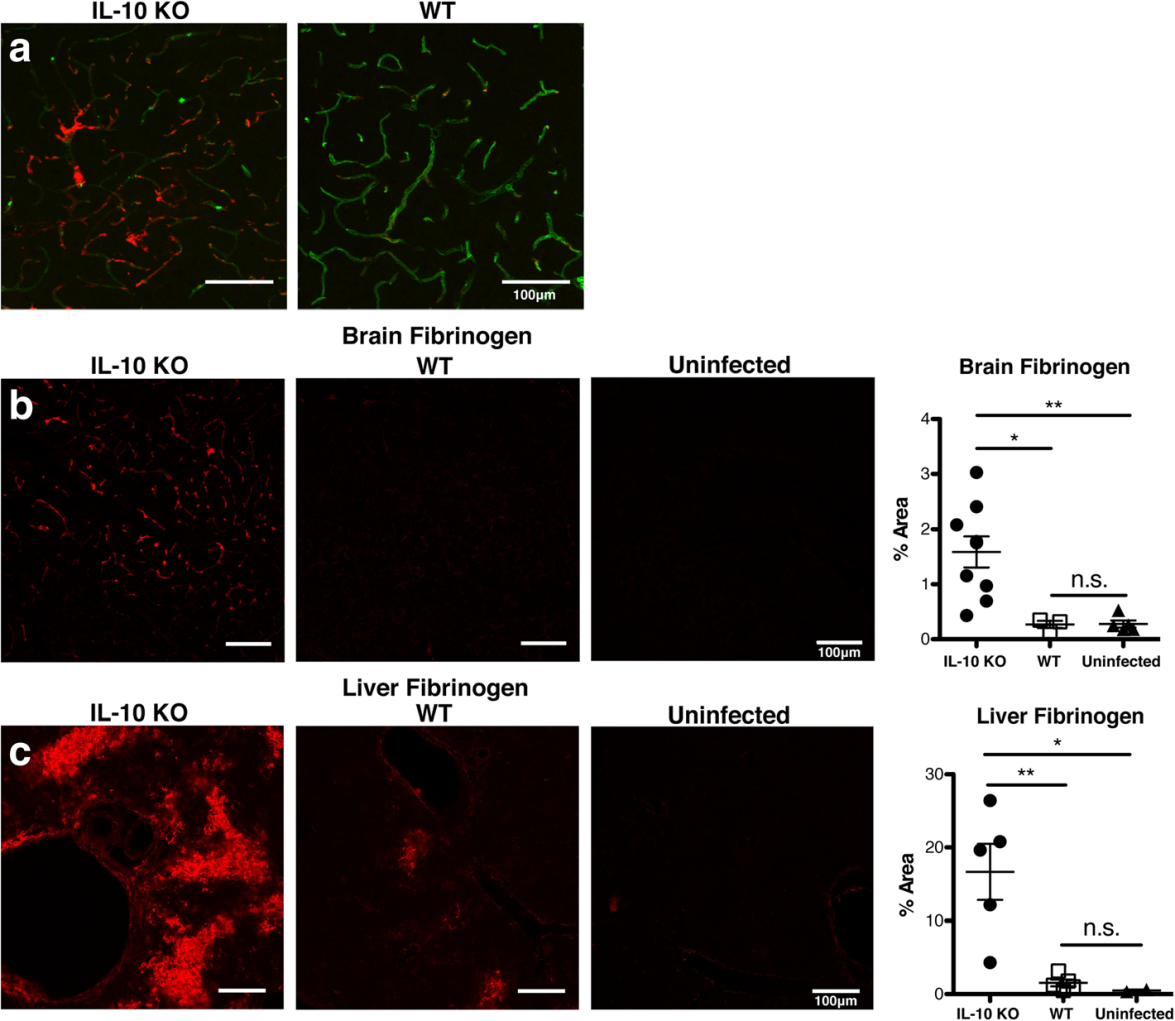
IL-10 KO mice have residual fibrin deposition in and around brain vasculature and increased liver fibrinogen. **a** Confocal images (× 20) showing immunofluorescent staining of fixed, frozen brain sections (30 μm) from *P. chabaudi*-infected IL-10 KO and WT mice (day 8 p.i., *n* = 4 mice/group). Fibrin (red) and tomato lectin (green, vascular endothelium). **b** Fibrin (red) was quantified by surveying 10 fields per brain section (× 10). Graph showing average percent area of fibrin-positive staining above threshold in each field. **c** Immunofluorescent staining (× 10) and quantitation of fibrinogen (red) in liver from infected IL-10 KO, WT, and uninfected controls (*n* = 4 mice/group). One-way ANOVA, followed by post hoc Bonferroni method, was used to determine statistical significance. *p < 0.05, **p < 0.01. Scale bar represents 100 μm

To investigate vascular abnormalities in *P. chabaudi*-infected IL-10 KO mice, we examined sagittal sections of perfused and fixed brain tissue for evidence of vascular leakage as indicated by extravascular fibrinogen at the peak of infection (day 8 post-infection). Brains from infection-matched, disease-resistant WT mice were used as controls (Fig. 1a). In addition to the expected sites of perivascular fibrinogen (evidence of fibrinogen leakage), we also found foci of fibrin(ogen) staining within the vascular lumen of brain blood vessels in IL-10 KO mice. As we had performed transcardial perfusion prior to sacrifice, this data is suggestive of intravascular thrombi. Quantification of fibrin(ogen) staining in the IL-10 KO mice showed an increase in the area of the brain with bright fibrinogen immunoreactivity (percent area of Alexa Fluor 568^+^ pixels, 10 fields/mouse) compared to infected WT, or uninfected, which were indistinguishable from each other (Fig. 1b). There was also a large increase in staining of fibrinogen in the livers of infected IL-10 KO compared to WT, which had some lighter staining as well that was not quantifiable over background levels in uninfected mice (Fig. 1c). This could potentially be due to an increase in fibrinogen production by the IL-10 KO mouse downstream of inflammation, as fibrinogen is an acute phase response protein [57]. However, while increased systemic production of fibrinogen is a risk factor for coagulation, it does not lead to clotting by itself [58]. However, an increase in liver fibrinogen production is not sufficient for accumulation of fibrin, which is triggered by the coagulation cascade [57, 58].

**Fig. 2:**
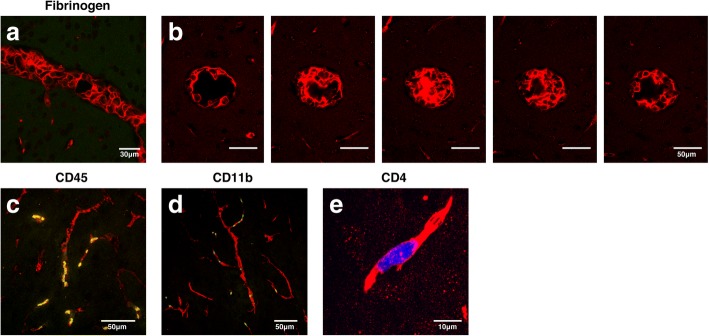
Vascular congestion in IL-10 KO mice with malaria includes thrombi-containing monocytes and T cells. Immunofluorescent staining of fixed, frozen brain sections (30 μm) from *P. chabaudi*-infected IL-10 KO mice (day 8 p.i., *n* = 4 mice). **a** Confocal images (× 40) of IL-10 KO brain stained for fibrin (red). **b** Successive single-plane confocal images (× 40) of a 30-μm z-stack showing complete occlusion of a large vessel with residual fibrinogen (red). **c** Immunofluorescence staining of IL-10 KO brains showing fibrin staining of blood vessels (red) and leukocytes expressing CD45 (green, × 60) and **d** CD11b (green, × 40). **e** CTV^+^ CD4 T cells (blue) from infected IL-10 KO mice were adoptively transferred into infection-matched IL-10 KO (day 7 p.i.) recipients 3.5 h before sacrifice. Frozen brain sections (day 7 p.i.) were stained for fibrin (red). Max intensity projection of a 30-μm z-stack (× 240) displayed from brain tissue of IL-10 KO mice co-stained with WT control samples (*n* = 3–4 mice per group). Scale bars represent 30 μm (**a**), 50 μm (**b**–**d**), or 10 μm (**d**)

Studies of both human CM and murine experimental cerebral malaria (ECM) have documented congestion of the brain and retinal vasculature, but the role of thrombi in reduced blood flow is not clear. By imaging through 200 μm of tissue, we found that both large and small vessels retain intravascular fibrin(ogen) (Fig. 2a), often to the point of complete occlusion of the vascular lumen (Fig. 2b), reminiscent of thrombosis. The coagulation cascade leads to cleavage of fibrinogen into fibrin during the formation of a clot [59]. The polyclonal antiserum used to detect fibrinogen here also detects fibrin and other degradation products of fibrinogen [60, 61]. Therefore, we interpret this staining pattern to represent fibrin clots. The appearance of sherical gaps in fibrin staining led us to hypothesize that in addition to red blood cells and platelets, immune cells could be retained within the thrombi of congested vessels. In order to identify them, we stained IL-10 KO brains for the pan-leukocyte marker, CD45 (Fig. 2c), and the monocyte marker, CD11b (Fig. 2d). Staining showed that many, but not all, CD45^+^ and CD11b^+^ leukocytes are contained within areas of residual fibrinogen staining. We previously quantified CD11b^+^ cells within the brains of *P. chabaudi*-infected IL-10 KO mice using flow cytometry. In that analysis, we showed that the CD11b^+^ cells were also Ly6C^+^, indicating that they are inflammatory monocytes [16]. There was a large and significant increase in cerebral Ly6C^hi^ inflammatory monocytes in IL-10 KO compared to that in infected WT mice, while a Ly6C^int^ population of resident macrophages was not increased.

We were also interested to see if CD4 T cells, the primary producers of IL-10 in this infection, were also found localized with fibrin(ogen) in the vessels. Therefore, CD4 T cells (CellTrace Violet^+^) from IL-10 KO mice 7 days post-infection (p.i.) were adoptively transferred into infection-matched IL-10 KO recipients, which underwent transcardial perfusion and brain tissue collection 3.5 h later. Transferred CD4 T cells were indeed identified in the brain, and often within a fibrin(ogen)^+^ clot (Fig. 2e). While the number of leukocytes is not large, activated leukocytes have the potential to promote activation of the neuroglial cells surrounding the vasculature, namely, astrocytes. Therefore, we next tested brain sections from infected IL-10 KO animals for astrogliosis.

### Inflammatory cytokine TNF induces astrocyte activation in clusters near thrombotic cerebral vasculature in IL-10 KO mice with malaria

**Fig. 3:**
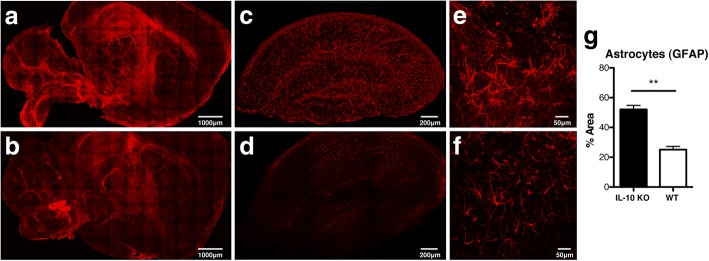
Increased astrocyte activation in IL-10 KO mice with malaria. Mice were infected with *P. chabaudi* and sacrificed 8 days post-infection. Thick sagittal brain sections (2 mm) were CLARITY-processed, optically cleared, stained with GFAP (red), and imaged by two-photon confocal microscopy. **a**, **c**, **e** IL-10 KO and **b**, **d**, **f** WT brains from the peak of *P. chabaudi* infection (day 8 p.i., *n* = 5 mice/group). **a**, **b** Single fields of the entire tissue section (× 10) stitched together. **c**, **d** Hippocampus of the thick brain section is masked for increased resolution and quantitation in **c** IL-10 KO and **d** WT animals (*n* = 3 mice/group). **e**, **f** Representative high-resolution image (× 25) of astrocytes from the hippocampus showing the **e** IL-10 KO and **f** WT control brains. **g** Quantification of the percent area of astrocyte staining above threshold in the hippocampal formation of the *P. chabaudi*-infected IL-10 KO and WT brains. Number of fields for IL-10 KO (*n* = 15) and WT (*n* = 9). Scale bars represent 1 mm (**a**, **b**), 200 μm (**c**, **d**), and 50 μm (**e**, **f**). Student’s *t* test was used to determine statistical significance. **p < 0.01

As astrocytes play an important role in maintaining the integrity of the BBB, including in the context of experimental cerebral malaria [62], we analyzed the extent of astrocyte activation in IL-10 KO mice infected with *P. chabaudi*. In order to visualize extensive activation of astrocytes, we utilized CLARITY followed by optical clearing, a tissue processing technique which removes relatively opaque lipids, transforming thick sagittal brain sections (2 mm) to make them optically transparent. This process diminishes excess light scattering during image acquisition by confocal or two-photon microscopy, allowing for increased imaging depth beyond that possible in unprocessed tissue. The ability to obtain image stacks over the full 2 mm thickness combined with image stitching allowed for image acquisition of the entire thick sagittal section. Whole brain sections stained for glial fibrillary acidic protein (GFAP), which is upregulated on activated astrocytes, were imaged to determine the extent of astrocyte activation in susceptible IL-10 KO mice (Fig. 3a, c, e) and resistant WT animals (Fig. 3b, d, f). A higher GFAP signal was observed in multiple areas of the IL-10 KO brain compared to WT, including the hippocampus, thalamus, and caudate putamen, suggesting astrocyte activation via increased production of inflammatory cytokines (Fig. 3a, b). While GFAP is expressed on most astrocytes, even in uninfected animals, the level of expression is significantly lower than on activated astrocytes [63]. Interestingly, there was little GFAP signal in the cortex, a result that is consistent with findings in human CM autopsy [20]. For quantitation of astrogliosis, we focused our analysis on the hippocampal formation (Fig. 3c, d), as a representative region in which astrogliosis was evident. This region is amenable to being isolated from other regions by image processing due to its well-defined margin and thus allowed for comparison of GFAP^bright^ cells in the full volume of the hippocampal region in each section. As shown in high-resolution 3D micrographs (Fig. 3e, f), in addition to upregulation of GFAP, astrocytes in IL-10 KO mice showed distinct morphological changes, appearing hypertrophied and with more processes compared to infected WT. The GFAP^bright^ fraction of the hippocampal formation in infected IL-10 KO mice was significantly increased compared to WT mice (Fig. 3g). While elevated serum ammonia from potential liver damage can activate astrocytes [64], there was no significant difference in ammonia production between WT and IL-10 KO mice (Additional file 2: Figure S2). As inflammation, or vascular damage, can also lead to astrocyte activation, we next investigated whether vascular congestion and astrocyte activation occurred in close proximity.

**Fig. 4:**
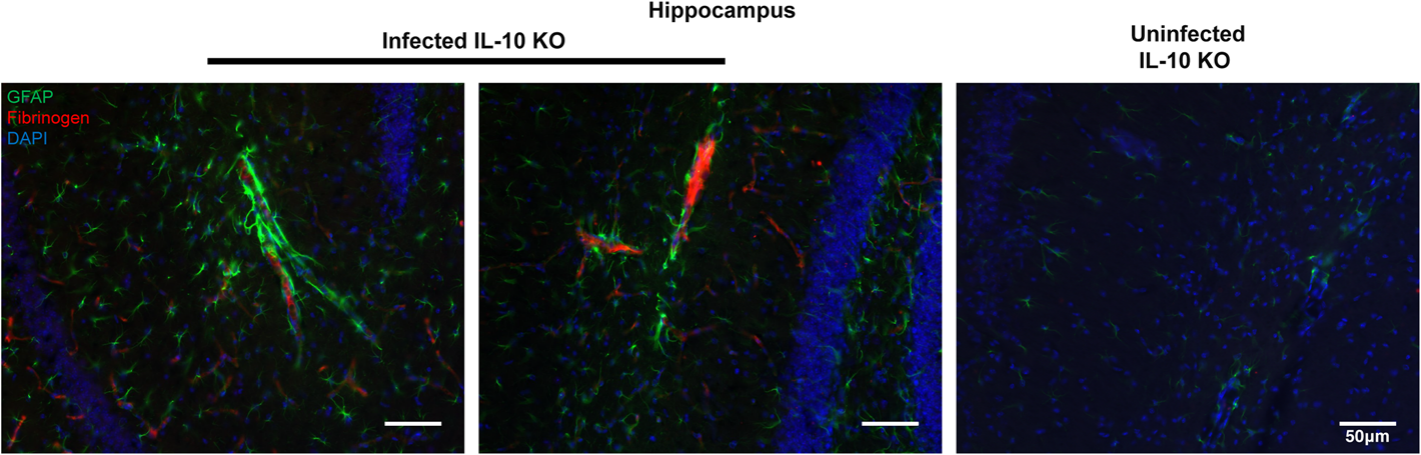
Activated astrocytes cluster along thrombus-containing brain vasculature. IL-10 KO mice were either infected with *P. chabaudi* and sacrificed 8 days post-infection or used as uninfected controls. Representative epifluorescence images (× 20) of the hippocampal formation in cryosections (30 μm) from infected (day 8 p.i.) IL-10 KO brains (left, middle) and uninfected IL-10 KO brains (right) immunostained for GFAP (green), fibrinogen (red), and DAPI (blue). IL-10 KO mice were co-stained with WT control samples (*n* = 5–6 mice per group). Scale bars represent 50 μm

To investigate the potential connection between vascular congestion and astrocyte activation, we performed immunofluorescent staining of peak-infected (day 7 p.i.) and uninfected IL-10 KO brains for fibrin(ogen) and astrocyte activation. In the hippocampal formation, we observed an increase in residual fibrin(ogen) staining in the infected IL-10 KO brains compared to WT (Fig. 4). Interestingly, the astrocytes showed an increase in GFAP staining and polarity and were more frequently found in contact with fibrin-containing vessels in infected IL-10 KO brains compared to infected WT and uninfected IL-10 KO controls (Additional file 3: Figure S3). However, it was noted that not all areas with residual fibrin staining were located near highly activated astrocytes. Uninfected mice showed neither residual fibrinogen deposition, nor an increase in GFAP immunoreactivity. Having established a link between microvascular congestion characterized by fibrin staining and astrocyte activation in this hyper-inflammatory response, we next sought to determine the role that inflammatory cytokines play in this process.

Immunopathology in IL-10 KO mice infected with *P. chabaudi* is generated by the hyper-inflammatory cytokine response generated in the absence of this regulatory cytokine primarily made by T cells [32]. Neutralizing TNF is known to improve survival and also improve all measures of symptomatic pathology, while *Ifngr1* deficiency in IL-10 KO mice improves survival [14, 31]. Importantly, neutralizing the other major regulatory cytokine, transforming growth factor-β, increases mortality of the IL-10 KO to 100%, suggesting that the balance of inflammatory and regulatory cytokines in the immune response to malaria infection determines the lethality of *P. chabaudi* in IL-10 KO mice [14]. However, the role of TNF in brain pathology, including its behavioral results, has not yet been investigated in this model. As an indication of brain pathology, we used a semi-quantitative *P. chabaudi*-specific SHIRPA health assessment abbreviated from one we previously described [16]. We have now identified a smaller set of behavioral symptoms, described in the “Methods” section, that specifically change at the time that IL-10 KO mice begin to succumb to infection. The SHIRPA screen was highly predictive of outcome, as the SHIRPA scores of mice that died during infection were significantly lower than those of mice that survived (Additional file 1: Figure S1). In addition, we were able to use the abbreviated SHIRPA to identify animals predicted to succumb to hyper-inflammatory experimental cerebral malarial disease. Any *P. chabaudi*-infected IL-10 KO mouse that scored below 17, out of a maximum of 22, on the abbreviated SHIRPA screen before day 9 post-infection had a statistically significant chance of succumbing to infection, with an odds ratio of 23.7 (95% CI 4.0–126.0, *χ*^2^ test), meaning they had almost 24 times more probability to succumb to disease. However, two out of 49 mice (4.1%) that were predicted to die actually survived. In addition, due to the speed of progression from undetectable morbidity to mortality, some animals (11/28, 39%) will die naturally without ever exhibiting a low SHIRPA score.

**Fig. 5:**
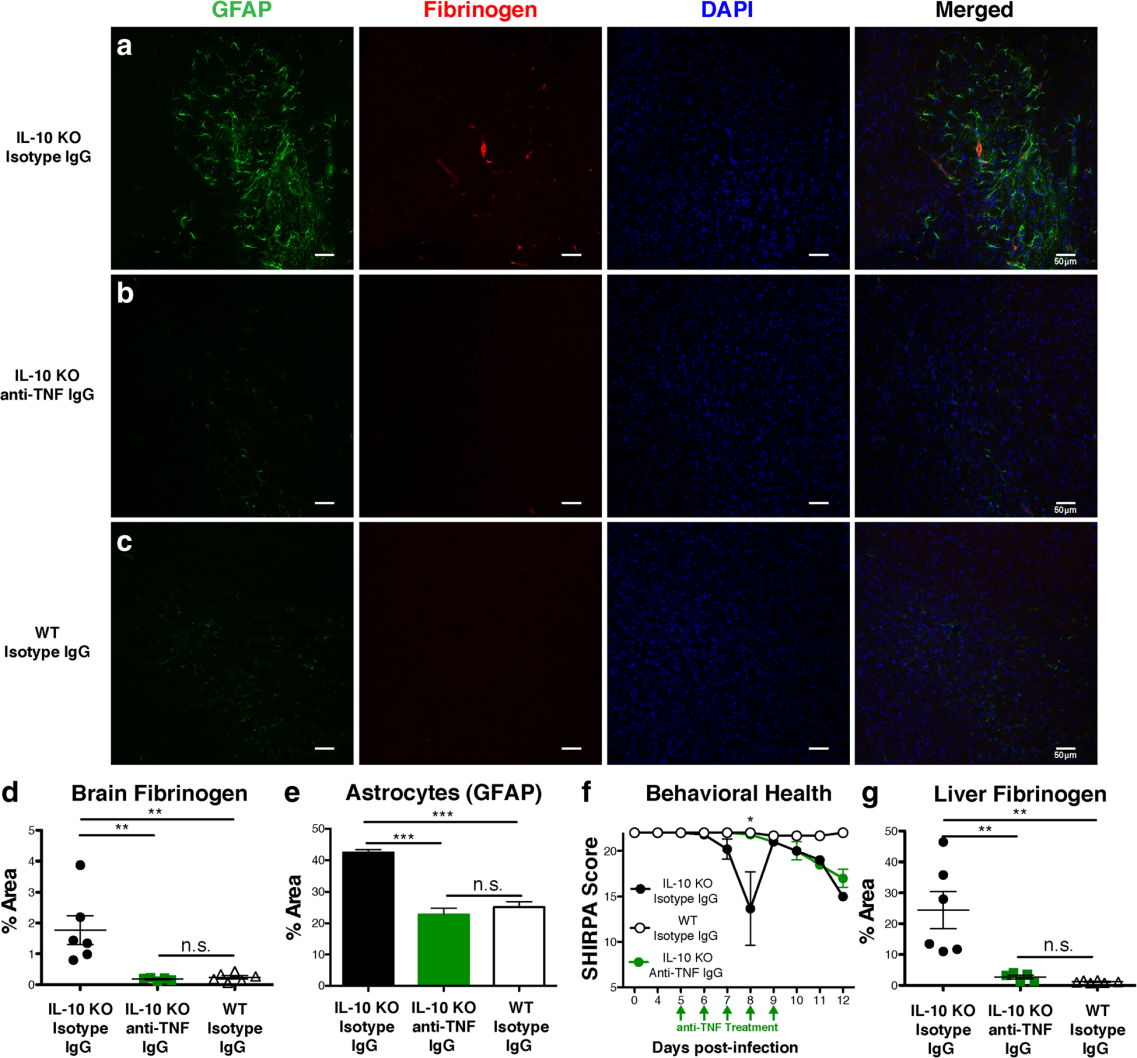
Anti-TNF antibody treatment prevents astrocyte activation and mortality in IL-10 KO mice with malaria. Mice were infected with *P. chabaudi* and followed throughout the acute phase of infection (day 12 p.i.) or sacrificed 8 days post-infection for immunofluorescent staining. One group of IL-10 KO mice received anti-TNF IgG treatment (*n* = 5), while another group of IL-10 KO mice (*n* = 5) and a group of WT mice received isotype IgG as control (*n* = 5). **a** Representative confocal images (× 20) of cryosections stained for astrocytes (GFAP; green) and fibrinogen (red) with DAPI (blue) in sagittal brain sections in anti-TNF antibody-treated IL-10 KO mice, **b** isotype IgG-treated IL-10 KO mice, **c** and isotype IgG-treated WT mice. **d** Brain fibrinogen and **e** GFAP staining for reactive astrocytes in the hippocampus were quantified by calculating the percent area per field of immunostaining above signal threshold. Ten fields per animal were assessed, with the graph showing the mean value per animal. **f** General behavior as measured by the abbreviated SHIRPA screen of anti-TNF antibody-treated (IL-10 KO, *n* = 5) and isotype IgG-treated (IL-10 KO, *n* = 5; WT, *n* = 5) mice infected with *P. chabaudi*. Green arrows represent the dosing schedule of either anti-TNF IgG or isotype control IgG. **g** Liver fibrinogen quantitation. Data shown is representative of two independent experiments (*n* = 9 total mice/group). One-way ANOVA, followed by post hoc Bonferroni method, was used to determine statistical significance. *p < 0.05, **p < 0.01, ***p < 0.001. Scale bars represent 50 μm

To test the role of TNF in neuroimmunopathology and astrocyte activation in this infection, we treated IL-10 KO mice with neutralizing anti-TNF antibody or isotype control antibody for 5 days (days 5–9 p.i.) [14]. To monitor for fibrinogen accumulation and astrocyte activation, mice were sacrificed at day 8 p.i., at the onset of severe disease, and brain tissue was stained for confocal microscopy. We observed an increase in astrocyte activation and increased residual fibrinogen in isotype-treated IL-10 KO animals (Fig. 5a), but neither of these changes were observed in the IL-10 KO group treated with neutralizing anti-TNF antibodies (Fig. 5b), similar to isotype-treated WT mice (Fig. 5c). These changes were significant, with a complete reduction in fibrinogen accumulation (Fig. 5d) and astrocyte activation (Fig. 5e). Furthermore, animals were protected from behavioral symptoms during anti-TNF treatment (Fig. 5f). Behavioral symptoms declined after treatment stopped, but we did not observe any late mortality. As expected, excess production of fibrinogen in the liver was also reduced by anti-TNF treatment (Fig. 5g). As anti-TNF blocks many components of the acute phase reaction besides coagulation, we proceeded with more specific tests for the importance of coagulation to hyper-inflammatory experimental cerebral malaria.

### Anticoagulant treatment eliminates early mortality and reduces glial cell activation in IL-10 KO mice with malaria

**Fig. 6:**
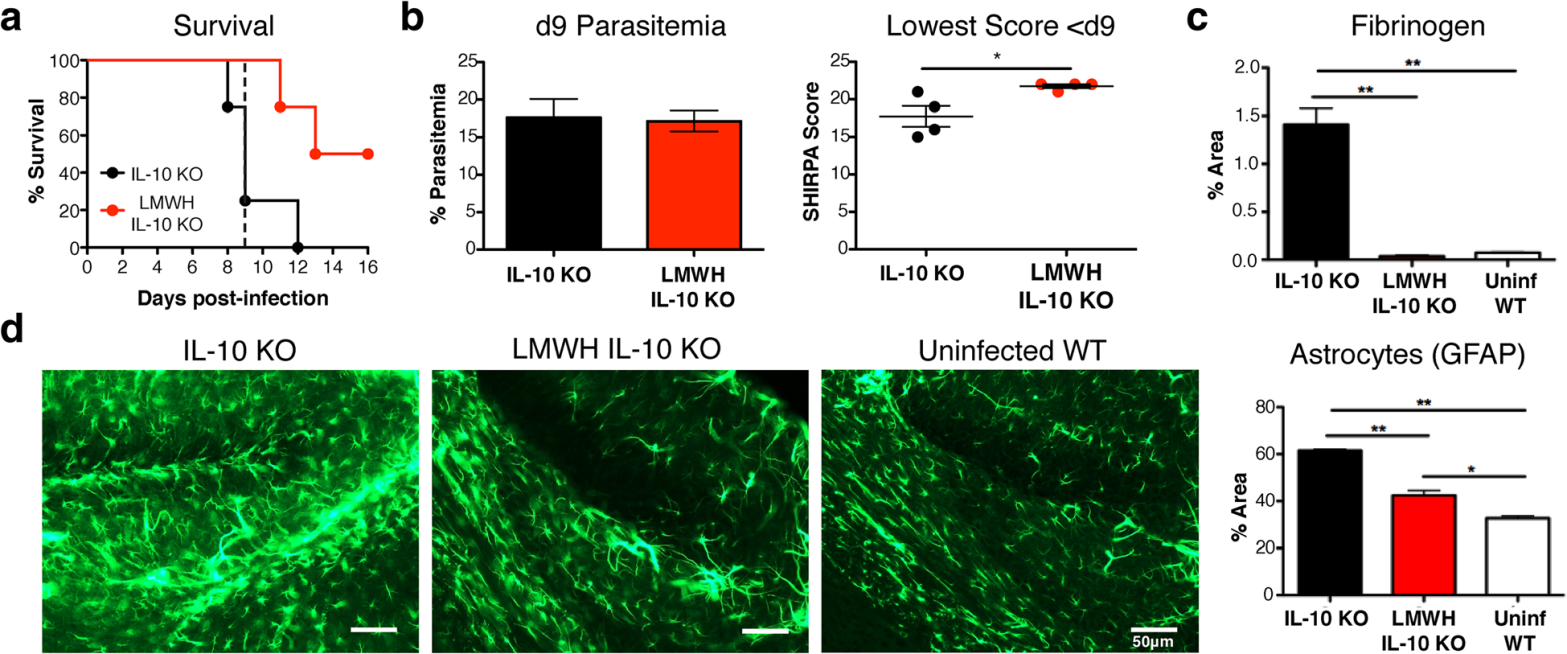
IL-10 KO mice are rescued from fatal neurologic disease with LMWH treatment. **a** Two groups of IL-10 KO mice (*n* = 4) were either treated with 1000 IU/kg (20 IU/dose) enoxaparin Na (ENO) i.p. twice a day (12 h apart) or given saline starting at day 4 post-infection until the middle of the anemic period of disease (day 12 post-infection). **b** Survival was monitored daily, and blood smears were collected on day 9 post-infection. Behavior was monitored daily using the abbreviated SHIRPA screen (*n* = 4 mice/group). **c** Fibrinogen quantification in the brains of untreated and LMWH-treated mice at the peak of infection (day 9 post-infection, *n* = 4 mice/group). **d** 30-μm brain hippocampus cryosections stained for astrocytes (GFAP, green). GFAP staining quantified by calculating the percent area per field of immunostaining above signal threshold. One-way ANOVA, followed by post hoc Bonferroni method was used to determine statistical significance. *p < 0.05, **p < 0.01. Scale bars represent 50 μm

To test the hypothesis that thrombi contribute to the fatal neurological phenotype of IL-10 KO mice infected with *P. chabaudi*, we treated infected IL-10 KO mice with the anticoagulant drug, enoxaparin sodium, a low-molecular weight heparin (LMWH), starting on day 4 post-infection through to the end of peak illness at day 12 post-infection, when all control animals had died. Mice were treated twice per day and monitored using the abbreviated SHIRPA screen. Blood smears were also collected on day 9 post-infection to monitor parasite burden. Strikingly, LMWH treatment of IL-10 KO mice rescued them from fatal neurologic disease before day 9 post-infection (Fig. 6a). However, LMWH-treated IL-10 KO mice were still susceptible to delayed mortality, as two out of four ENO-treated mice (50%) died after day 9 post-infection. This may represent death from severe anemia that typically presents after the peak of *P. chabaudi* infection [65]. The differential mortality between treatment groups was not due to differences in parasitemia at the peak of infection on day 9 p.i., while behavioral scores were significantly improved with LMWH treatment (Fig. 6b). As a control to assure the quality of the treatment, we quantitated fibrinogen deposition in the brains of treated animals and confirmed that LMWH eliminated thrombi completely (Fig. 6c). Strikingly, we found that astrogliosis was significantly reduced by anticoagulant treatment, though not to the levels seen in uninfected animals (Fig. 6d). In conclusion, LMWH treatment decreased astrocyte activation and intravascular fibrin clotting, suggesting that thrombi in cerebral vasculature play a critical role in astrogliosis and lethal pathology from malaria without affecting parasitemia.

**Fig. 7:**
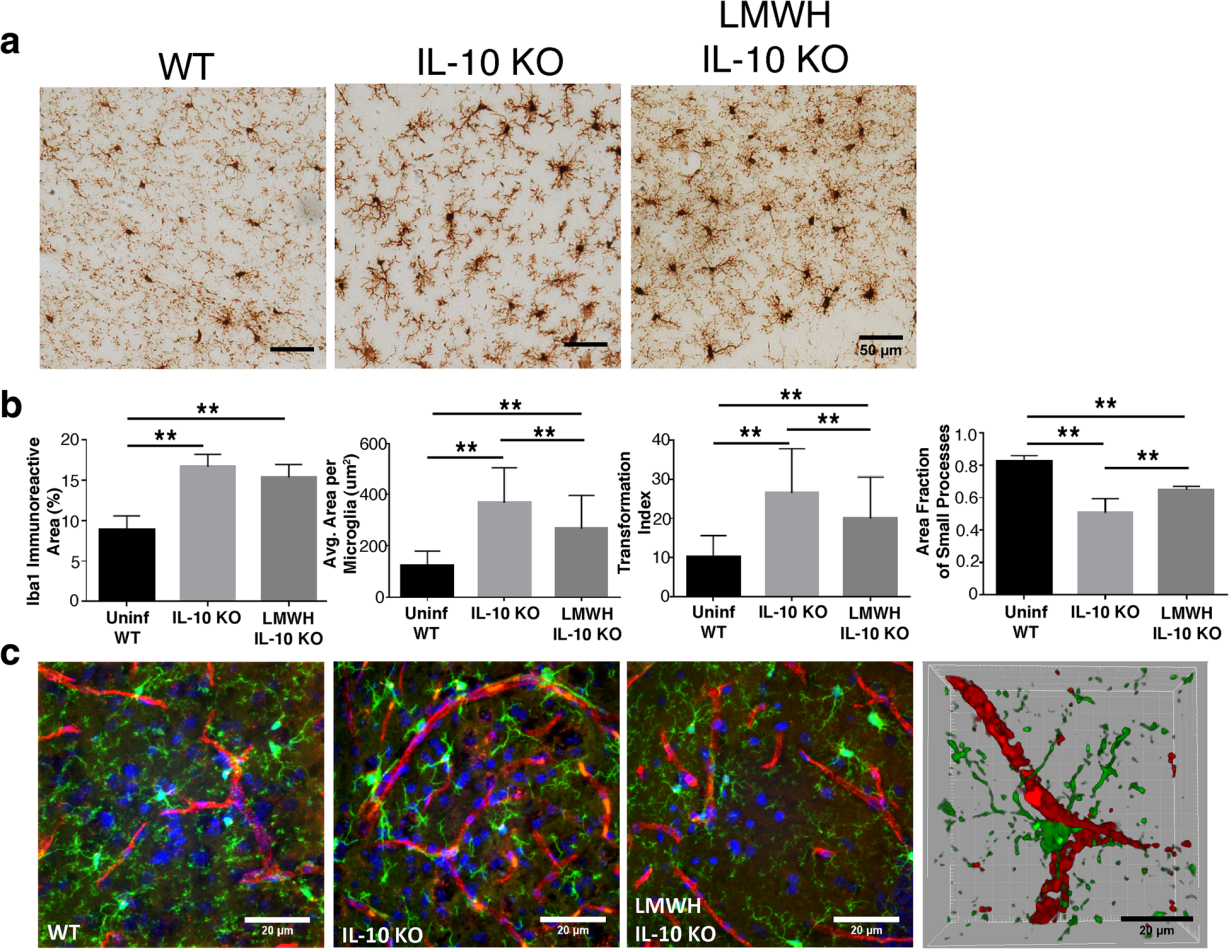
Microglia changes in IL-10 KO mice infected with *P. chabaudi*. **a** Representative images of day 8 p.i. WT, IL-10 KO, and LMWH-treated IL-10 KO mice (*n* = 4 mice/group) 30-μm brain cryosections stained with anti-Iba-1 antibodies and visualized using DAB. **b** Quantitative analysis of microglia morphology in WT, IL-10 KO, and LMWH-treated IL-10 KO mice using ImageJ software. **c** Immunofluoresence imaging of microglia (Iba-1-Alexa 488, green), endothelial cells (CD31-Alexa 567, red), and nuclei (DAPI, blue) in 30-μm brain cryosections from WT, IL-10 KO, and LMWH-treated IL-10 KO mice during the peak of infection. Right, 3D reconstruction showing the spatial orientation of microglia cells in relationship to microvasculature in a *P. chabaudi*-infected IL-10 KO mouse. One-way ANOVA, followed by post hoc Tukey’s test, was used to determine statistical significance. **p < 0.01. Scale bars represent 20 and 50 μm

Microglia are important sentinels and potent amplifiers of inflammation within the CNS. In response to environmental cues and inflammatory stimuli, microglia become activated and undergo characteristic morphological changes. Therefore, we quantified both upregulation of Iba1, a marker of activation, and morphological changes characteristic of microglial activation in brain sections from either uninfected or *P. chabaudi*-infected mice on day 8 p.i. (Fig. 7a). We observed dramatic changes in the microglia in the IL-10 KO compared to WT, and we observed further changes in the anticoagulant-treated animals. To interpret these changes, we quantitated the extent of microglial activation in these images based on morphology. We used four quantitative assessments: (1) total immunoreactive area (% of total Iba1-positive pixels in a field); (2) average immunoreactive area per microglia; (3) transformation index, a measure of microglial ramification; and (4) area fraction of small processes, which is normalized to total immunoreactive area. The latter was done to capture differences in small/fragmented processes, as small processes were not observed in the IL-10 KO group, while they were present in the LMWH group, although not as numerous as the WT group (Fig. 7b). The last graph, therefore, shows how much Iba1-reactive area each group has with respect to the area occupied by microglia soma, which was significantly lower in the untreated IL-10 KO group. We interpret this to mean that activated microglia retract their dendrites, which then appear thicker, as opposed to the thinner processes that cover more three-dimensional area in homeostasis. All of these measures suggest that microglial activation is reduced, but not back to homeostatic levels, by LMWH treatment, similar to what we found for astrogliosis above.

In order to determine the relative localization of activated microglia and cerebral vasculature, immunofluorescent staining was performed on microglia (Iba1) and CD31^+^ blood vessels (Fig. 7c). We observed increasing microglial polarity and thickening of dendrites in IL-10 KO animals, with decreased numbers of small processes in the microglia of untreated IL-10 KO mice. The localization of microglia near vessels in infected animals is clearly seen when viewed as a 3D stack. Enumeration of the number of microglia that interacted with a blood vessel, defined as either body or process on the blood vessel, indicated 79% of glia interacted with a vessel in the KO group vs. 54% in the WT (*p* < 0.05), and while mean value for LMWH-treated IL-0 KO mice was 69%, it was not statistically significant from either KO or WT. The morphological changes in infected IL-10 KO mice show significant changes in microglial activation state, suggestive of increased intracranial inflammation. Interestingly, all features of activation show significant improvement towards homeostasis after clearance of thrombi following LMWH treatment. Therefore, these findings demonstrate a critical role of inflammation-driven coagulation in experimental cerebral malaria pathology.

## Discussion

The presence of peripheral immune cells adherent within the vasculature in mouse models of CM and in brain vessels on autopsy of cerebral malaria patients [66] suggest that such cells play an important role in mediating neuropathology [67]. Current paradigms to explain CM pathogenesis support an important role for inflammation in the generation and amplification of neuropathology but do not explain the derivation of these cytokines in the brain. The derivation and contribution of cerebral thrombi to CM pathology is also poorly understood. The vascular findings in this study suggestive of pervasive (Fig. 1) and complete (Fig. 2) blockade of the vasculature by inflammation-induced thrombi are striking. These abnormalities have not been described in *P. chabaudi* infection before. Coagulation is clearly of major relevance for our understanding of pathological mechanisms in cerebral malaria [21, 58, 68]. Potentially pathogenic serum levels of both pro- and anticoagulation proteins have been documented in human CM [69, 70]. Systemic inflammation has also recently been shown to contribute to intravascular clotting via mechanisms involving neutrophils and monocyte interaction with platelets in CM [71, 72], linking inflammation and clotting, which in turn promote sequestration. Recent studies also show that the anticoagulation endothelial protein C receptor (EPCR) may bind the parasite and be downregulated, thus promoting clotting and suggesting a mechanism for the induction of coagulation by *P. falciparum* sequestration [45, 73]. Interestingly, studies point to the bi-directional amplification of the clotting cascade and inflammation suggesting an important intersection that is likely to be crucial to pathology in CM [58].

The data presented here confirm that inflammatory cells within the vasculature can drive both clot formation and activation of cells in the brain parenchyma in the absence of local parasite adhesion. Studies of *Plasmodium berghei* (ANKA) (PbA) infection have established the importance of the inflammatory response in the development of neurocognitive dysfunction [74–76]. PbA infection shows pathogenic immune cell accumulation in cerebral blood vessels as a result of inflammatory TNF and IP-10 secretion [77, 78] and intercellular adhesion molecule-1 (ICAM-1) on the vascular endothelium [79]. PbA infection has also been shown to induce astrocyte activation and degeneration near sites of monocyte vascular adhesion [62, 80]. However, the signals leading to the breakdown of local astrocyte barrier function in malaria have not yet been defined. The activation of astrocytes is a feature of many neurological diseases, including cerebral malaria [81, 82]. Our results demonstrate a causal link between hyper-inflammation, hyper-coagulation, glial cell activation, and mortality (Figs. 3 and 4). Gliosis across multiple areas of the brain was observed in infected IL-10 KO mice, with astrocytes and microglia associating highly with the vasculature compared to the WT group—yet both microglial and astrocyte gliosis were significantly reduced upon LMWH treatment, indicating this direct link.

This is important because resolution of CM in African children and Asian adults can be resistant to anti-malarial drug treatment, suggesting that parasite alone does not cause the full cerebral malaria syndrome. Furthermore, it is not yet clear how parasite adhesion alone drives the neuropathology evident from patient symptoms [83]. However, because of the overlap of inflammation with parasite-dependent factors, determining the independent contributions of each presents an ongoing challenge to investigators. The impact of parasite adhesion to the vascular endothelium on coagulation, vascular integrity, and congestion has been shown in in vitro endothelial cultures and animal models of cerebral malaria [19, 43, 67, 84, 85]. Sequestration is seen in most fatal pediatric and adult CM cases [20, 21] and is used as a critical hallmark of disease. We chose to study the role of inflammatory cytokines in isolation from the potential contribution of sequestration using an inflammation-induced cerebral malaria model. The results confirm that inflammation can cause many of the pathological changes seen in CM, though not all.

In this study, we show that both the congestion phenotype associated with intravascular clotting and astrocyte activation can be reversed via neutralization of TNF (Fig. 5), or anticoagulant therapy (Fig. 6). Serum TNF concentration correlates with severity of human malaria [86]. However, TNF blockade has thus far proven ineffective in preventing death in childhood cerebral malaria [87, 88]. As different reagents displayed differential effects, the timing, dose, or precise antigenic specificity of treatments may yet be improved for adjuvant therapy. Strikingly, these data also show that fatal neurological disease in IL-10 KO mice is dependent on intravascular coagulation, as it can be prevented by LMWH treatment (Fig. 6). This demonstrates a central role for thrombi in driving the disease mortality and promoting neuropathology in *P. chabaudi* infection of IL-10 KO mice. As anti-TNF and anticoagulants have similar effects in this model, it is likely that cytokines and the coagulation cascade promote each other, as in other systems. Despite the WHO recommendation against the use of heparin since 1984, citing excessive bleeding [89], there are several clinical trials showing significant beneficial effects of anticoagulant usage on mortality and length of coma in human CM [49, 50, 90, 91]. Selection of treatments with relatively moderate anticoagulation activity is likely essential to achieving therapeutic goals while avoiding hemorrhagic complications. LMWH, as the name implies, involves only the activity of the smaller heparin proteins, which act with higher specificity on factor Xa, exhibit less thrombin inhibition, and produce a more reliable therapeutic profile. Our studies show that LMWH treatment is protective within the context of hyper-inflammatory cerebral malaria and prevents intravascular thrombi formation in the brains of mice exhibiting behavioral dysfunction (Fig. 6). This is particularly important in that both astrocyte and microglial activation were dependent on this coagulation event to some degree (Figs. 6 and 7). Activation of microglia has been shown to be an important component of neuroinflammation and behavioral dysfunction associated with PbA infection [92–94]. Widespread microglial activation, not always restricted to areas of parasite sequestration, has also been identified in cases of human CM [95, 96]. However, these findings are novel in the context of *P. chabaudi* infection. Furthermore, the spatial relationship of intravascular coagulation with glial cell activation is also previously unknown in any malaria infection and should be examined in human CM autopsy samples.

Efforts to manipulate the inflammatory response and clotting cascade have provided mixed results in clinical trials to date [97–99], highlighting the importance of understanding the interactions between various arms of the host response within the pathogenesis of cerebral malaria. In summary, our experiments support the importance of intravascular coagulation and leukocytes producing inflammatory cytokines in malaria-induced cerebral pathology. The activation of surveilling microglia and vascular/neuronal-supportive astrocytes downstream of systemic inflammation could promote the generation of neuropathology secondary to malaria infection. Identification of both T cells and monocytes within fibrin clots suggests a new working model where inflammatory cells promote cerebral damage even from their localization within the cerebral vasculature. It is possible that leukocytes within the structure of intravascular thrombi serve to amplify pathological inflammatory cytokines leading to immunopathology in the brain. These data demonstrate the interaction of the anti-parasitic and hemostatic elements of host defense, promoting a new appreciation of the interplay between mechanisms important for development of fatal cerebral malaria.

## Conclusions

Our study has identified intravascular thrombi within the cerebral vasculature during severe *P. chabaudi* infection and showed that they contribute to lethal immunopathology. Furthermore, vascular congestion with an accumulation of leukocytes is spatially associated with astrocyte and microglial activation in this model, with the former being driven by TNF. The most striking finding is that dissipation of these inflammatory foci within fibrin-rich thrombi by LMWH treatment leads to a significant decrease in early lethal pathology. These findings begin to define the parameters of inflammation in the brain during cerebral malaria, and the downstream pathology linked to hyper-inflammation. Previously, findings of cytokine gene linkage to CM were understood in terms of increasing parasite binding within the capillary bed. Our findings demonstrate that inflammatory cytokines contribute both pathogenic coagulation and activation of sentinel glia in the brain parenchyma, which are capable of causing neurological sequelae, even in the absence of localized sequestration, although to a lesser degree than more virulent parasites. These findings, therefore, contribute to the current understanding of the etiologies of cerebral pathology and neurovascular abnormalities in malaria infection. While the effectiveness and safety of this approach must be validated, the positive effect of anticoagulants could inform development of future adjunctive therapy for CM patients.

## Additional files

Additional file 1: **Figure S1.** IL-10 KO mouse behavioral scores are predictive of outcome during *P. chabaudi* Infection. Left: representative experiment showing SHIRPA scores of infected male IL-10 KO mice grouped by eventual outcome (survived = blue, *n* = 6; died = black, *n* = 5). Right: graph of the lowest abbreviated SHIRPA score in individual mice before day 9 post-infection with infected IL-10 KO mice grouped according to outcome. Showing concatenated data from multiple experiments (*n* = 48). Error bar represents SEM, ***p < 0.001, Wilcoxon signed rank test. (TIF 326 kb)

Additional file 2: **Figure S2.** IL-10 KO mice ammonia levels are not elevated above WT during *P. chabaudi* infection. WT and IL-10 KO mice were infected with *P. chabaudi* and monitored during the peak of infection. WT mice were sacrificed at the peak of infection (day 10 p.i.) and IL-10 KO mice upon severe morbidity as determined via SHIRPA score. Organ and plasma ammonia levels were measured using a colorimetric ammonia assay. (TIF 218 kb)

Additional file 3: **Figure S3.** *P. chabaudi*-infected IL-10 KO mice show astrocyte association with thrombi. Left, normalized astrocyte-thrombus association ratio. Right, representative confocal images of experimental groups stained for astrocytes (green) and fibrinogen (red). *N* = 3–5 mice/group. Error bar represents 30 μm. (TIF 12464 kb)

## Abbreviations

3D: Three-dimensional
BBB: Blood-brain-barrier
CM: Cerebral malaria
CNS: Central nervous system
CXCR3: C-X chemokine receptor 3
DIC: Disseminated intravascular coagulation
ECM: Experimental cerebral malaria
GFAP: Glial fibrillary acidic protein
i.p.: Intraperitoneal
ICAM-1: Intracellular adhesion molecule-1
IFN-γ: Interferon gamma
IHC: Immunohistochemistry
IL-10 KO: IL-10-deficient
iRBCs: Infected red blood cells
MHC-II: Major histocompatibility complex class II
PbA: *Plasmodium berghei* (ANKA)
SHIRPA: SmithKline Beecham, Harwell, Imperial College, Royal London Hospital Phenotype Assessment
TNF: Tumor necrosis factor
WT: Wild-type, C57Bl/6J
