# Supplementary figures and images for: Elimination of intravascular thrombi prevents early mortality and reduces gliosis in hyper-inflammatory experimental cerebral malaria
Source: J Neuroinflammation. 2018 Jun 4;15:173. doi: 10.1186/s12974-018-1207-4 (PMC5987620; doi:10.1186/s12974-018-1207-4)

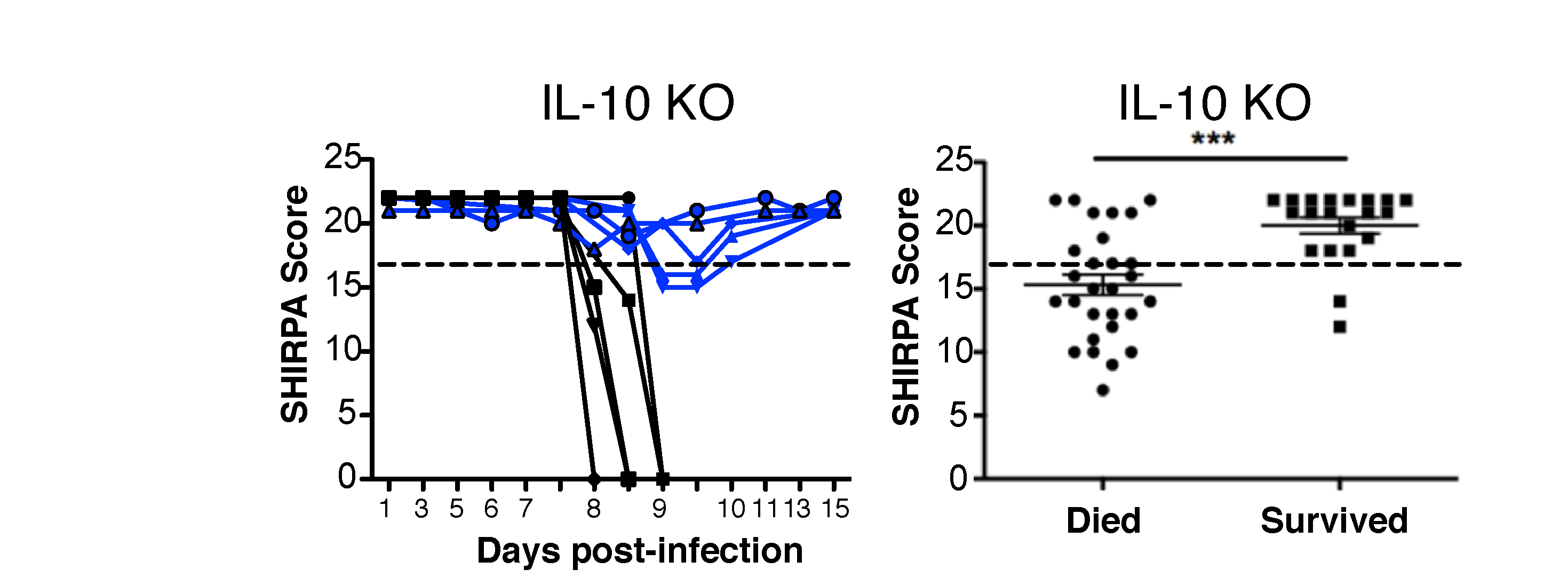

Supplement: Supplementary file 1 — Figure S1. IL-10 KO mouse behavioral scores are predictive of outcome during P. chabaudi Infection. Left: representative experiment showing SHIRPA scores of infected male IL-10 KO mice grouped by eventual outcome (survived = blue, n = 6; died = black, n = 5). Right: graph of the lowest abbreviated SHIRPA score in individual mice before day 9 post-infection with infected IL-10 KO mice grouped according to outcome. Showing concatenated data from multiple experiments (n = 48). Error bar represents SEM, ***p < 0.001, Wilcoxon signed rank test. (TIF 326 kb) [file 12974_2018_1207_MOESM1_ESM.tif]

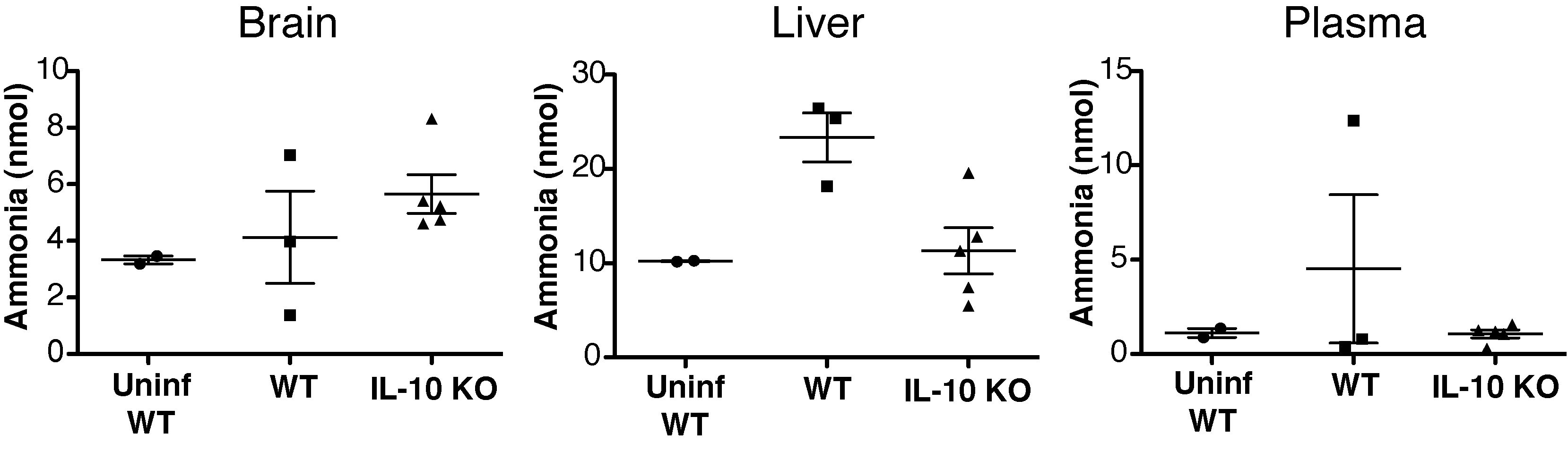

Supplement: Supplementary file 2 — Figure S2. IL-10 KO mice ammonia levels are not elevated above WT during P. chabaudi infection. WT and IL-10 KO mice were infected with P. chabaudi and monitored during the peak of infection. WT mice were sacrificed at the peak of infection (day 10 p.i.) and IL-10 KO mice upon severe morbidity as determined via SHIRPA score. Organ and plasma ammonia levels were measured using a colorimetric ammonia assay. (TIF 218 kb) [file 12974_2018_1207_MOESM2_ESM.tif]

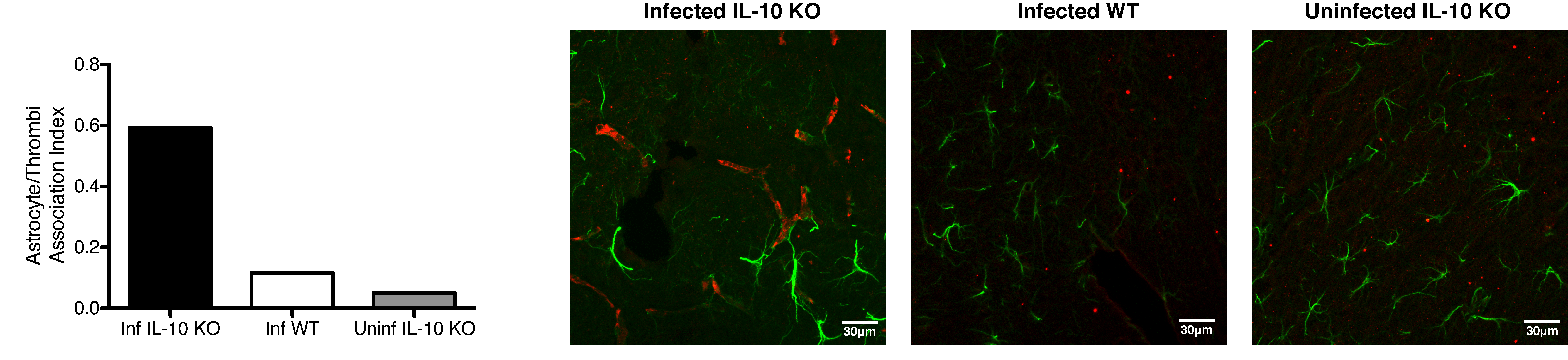

Supplement: Supplementary file 3 — Figure S3. P. chabaudi-infected IL-10 KO mice show astrocyte association with thrombi. Left, normalized astrocyte-thrombus association ratio. Right, representative confocal images of experimental groups stained for astrocytes (green) and fibrinogen (red). N = 3–5 mice/group. Error bar represents 30 μm. (TIF 12464 kb) [file 12974_2018_1207_MOESM3_ESM.tif]
